# Supplementary material for: Effectiveness of a family-centered method for the early identification of social-emotional and behavioral problems in children: a quasi experimental study
Source: BMC Public Health. 2011 Aug 9;11:636. doi: 10.1186/1471-2458-11-636 (PMC3175466; doi:10.1186/1471-2458-11-636)
Supplement: Additional file 1 — Appendix 1. Overview of the contents of the family-centered approach; the five domains and corresponding questions. (PDF file). [file 1471-2458-11-636-S1.PDF]

Appendix 1: Overview of the contents of the family-centered approach; the five domains and corresponding questions

1. Competence of the primary caretaker

- How do you like being a mother (of … children)?
- Does the situation correspond to what you expected?
- Do you feel uncertain or do you have any difficulties with certain aspects of care? If you have, what kind of aspects are these?
- To what extent do you have time for yourself or for other activities?
- How do you think your health is?

Summarizing: the competence of the parent can be concluded as…

2. Role of the partner

- How does your partner feel about having a child?
- To what extent does your partner contribute to the care of your child?
- To what extent are you satisfied with the contribution of your partner?
- To what extent do you and your partner agree on how to raise and care for children?
- What happens if you and your partner do not agree (about how to raise and care for children)?
- How is the relationship between you and your partner in general?
  (in case of no relationship: how do you feel about that?)
- What is the impact of having a child on your relationship?

Summarizing: the role of the partner can be concluded as…

3. Social support

- Who supports you emotionally in caring for your child?
- Who supports you in practical terms in caring for your child?
- Who advises you about caring for your child?
- To what extent do you manage with the support you receive?
- Are you familiar with ways to enlarge your social network?
- To what extent are you in need of contact with other mothers with babies?

- How would you define your relationship with your own parents?

Summarizing: the social support can be concluded as…

4. Perceived barriers or life events within the care-giving context of the child

- Have there been any life events the past year?

  If so: To what extent does this influence your contact with (name of the child)?

- How does the combination of work and child care services work for you?
- How is your financial situation?
- How is your housing situation?
- Are there any other circumstances that impact on your family?

Summarizing: the perceived barriers or life events within the care giving context can be concluded as…

5. Wellbeing of the child

- How is (name of the child) doing overall?
- How is (name of the child) developing on a social-emotional level according to you?
- How familiar are you with (name of the child)?
- How does (name of the child) respond to his/her environment?
- To what extent do you recognize different ways of crying?

Summarizing: the wellbeing of the child can be concluded as…
